# GH and IGF System: The Regulatory Role of miRNAs and lncRNAs in Cancer

**DOI:** 10.3389/fendo.2021.701246

**Published:** 2021-08-16

**Authors:** Cecilia Catellani, Gloria Ravegnini, Chiara Sartori, Sabrina Angelini, Maria E. Street

**Affiliations:** ^1^Department of Mother and Child, Azienda USL-IRCCS di Reggio Emilia, Reggio Emilia, Italy; ^2^PhD Program in Clinical and Experimental Medicine, University of Modena and Reggio Emilia, Modena, Italy; ^3^Department of Pharmacy & Biotechnology, University of Bologna, Bologna, Italy

**Keywords:** GH, IGF, miRNA, lncRNA, cancer

## Abstract

Growth hormone (GH) and the insulin-like growth factor (IGF) system are involved in many biological processes and have growth-promoting actions regulating cell proliferation, differentiation, apoptosis and angiogenesis. A recent chapter in epigenetics is represented by microRNAs (miRNAs) and long non-coding RNAs (lncRNAs) which regulate gene expression. Dysregulated miRNAs and lncRNAs have been associated with several diseases including cancer. Herein we report the most recent findings concerning miRNAs and lncRNAs regulating GH and the IGF system in the context of pituitary adenomas, osteosarcoma and colorectal cancer, shedding light on new possible therapeutic targets. Pituitary adenomas are increasingly common intracranial tumors and somatotroph adenomas determine supra-physiological GH secretion and cause acromegaly. Osteosarcoma is the most frequent bone tumor in children and adolescents and was reported in adults who were treated with GH in childhood. Colorectal cancer is the third cancer in the world and has a higher prevalence in acromegalic patients.

## GH and IGF System

Growth hormone (GH) and the insulin-like growth factor (IGF) system consists of several components: GH, and receptor (GHR), IGF1, IGF2, Insulin-like growth factor 1 receptor (IGF1R), Insulin Like Growth Factor 2 Receptor (IGF2R), IGF binding proteins (IGFBPs), and related proteases which interact with each other and regulate several metabolic processes and body growth (1).

GH, also known as somatotropin, is produced in the anterior pituitary under the control of growth hormone releasing hormone (GHRH) which induces its synthesis and release (2, 3). Ghrelin, a peptide produced in the stomach related with nutritional stimuli, promotes also GH secretion by somatotropic cells. Conversely, GH pulsatile secretion is inhibited by somatostatin (1). In addition, GH is secreted locally by extra-pituitary tissues including neural, immune, skeletal, gastrointestinal, embryonic and reproductive tissues, exerting autocrine and paracrine roles (4). Bone and liver are important target tissues of GH, where GH binds with high affinity to the extracellular domains of GHR. This interaction leads to the phosphorylation of the tyrosine residues of the Janus kinase-2 (JAK2) which mainly activates Signal Transducer and Activator Of Transcription (STAT), Rat sarcoma protein/Extracellular signal-regulated kinase (Ras/ERK) and phosphoinositide 3-kinase/Protein kinase B/AKT Serine/Threonine Kinase (PI3K/AKT) intracellular signaling pathways (5). In the liver, GH stimulates the production of IGF1 which is a peptide hormone, that in turn regulates GH release establishing a negative feedback loop (6). IGF1 and IGF2, which belongs also to the IGF family, are produced also by many other tissues besides the liver and IGF2 is produced independent of GH stimulation (7). IGF2 mainly regulates fetal development while IGF1 increases after birth throughout growth and reduces with aging. On the target cell surface, IGF1 binds IGF1R which auto-phosphorylates and activates a signaling cascade mediated by the phosphorylation of the Insulin receptor substrate (IRS) proteins and other effectors leading to the activation of Mitogen-activated protein kinase (MAPK) and PI3K/AKT signaling pathways. Conversely, IGF2 after binding to the IGF2R does not transduce any intracellular signal. Indeed, IGF2R sequesters IGF2 from the circulation preventing its binding to the IGF1R and facilitating IGF2 degradation (8). IGF2 can bind also to the IGF1R and to the insulin receptor isoform A (IR-A) exerting its mitogen effects which have been extensively studied especially in the context of colorectal cancer (CRC) as described in a recent review (9).

The bioavailability of both IGF1 and IGF2 is controlled by a superfamily of six IGFBPs (IGFBP 1-6) characterized by a higher affinity for IGFs than IGF receptors (10). Circulating IGFs are mainly associated with IGFBP-3 and acid labile subunit, forming a ternary complex which can dissociate allowing the IGF-IGFBP complex to pass through the vessels and reach target tissues (1). The molecular structure of IGFBPs contains two homologous domains (N-terminal and C-terminal) for the IGF binding and a linker domain, which is the site for post-translational modifications and proteolysis (11). IGFBP proteolytic cleavage, which modulates IGFBP levels, is mediated by proteases belonging to three main superfamilies: serine proteases, matrix metallopeptidases (MMP), and cathepsins (8).

The GH and IGF system have growth-promoting actions regulating cell proliferation, differentiation, apoptosis and angiogenesis (1, 12, 13). Furthermore, the GH and IGF system components are also locally produced by cancer cells and exert both autocrine and paracrine roles increasing the complexity of the global framework (2). However, an involvement of GH in the development of malignancies remains unclear. Recently, it has been speculated that GH may promote neoplastic growth by contributing to create a favorable microenvironment (14). This uncertainty remains an aspect of particular importance and interest within conditions requiring GH treatment (15–18). Among these studies, a possible increased risk mainly in bone cancer is hypothesized (16, 17) whereas conditions characterized by increased GH secretion such as pituitary adenomas (gigantism/acromegaly) have an increased risk of colon cancer (19, 20).

## MicroRNAs and Long Non-Coding RNAs Are Epigenetic Regulators of Gene Expression

MicroRNAs (miRNAs) are a family of small non-coding RNAs (19–25 nucleotides -nts) that can regulate a plethora of biological processes *via* the modulation of the expression of target genes at the post- transcriptional level (21). In particular, miRNAs bind to a specific sequence, usually localized in the 3’ untranslated regions (3’UTR) of their target mRNAs which can be either degraded or their translation can be inhibited based on sequence complementarity (22, 23). The first study identifying miRNAs as key players in human disease dates back to 2002, when Calin et al. showed that miR-15a/16-1 cluster was frequently deleted in chronic lymphocytic leukemia, and ascribed to these miRNAs a tumor suppressor role (24). After this, a growing number of reports have been published and the importance of miRNAs became clear. Tumors have a clear miRNA signature, the so-called “miRNome”, which is specific for each tumor tissue and is often associated with clinical-pathological features of the tumors (25).

MiRNAs act in a very specific, context-dependent manner, and may have a dual role both as onco-miR and tumor-suppressor miR (26). For example, miR-221 and miR-222 are important in gastrointestinal stromal tumors where they target the driver oncogene KIT, functioning as tumor suppressors. However, in other solid tumors, including glioblastoma, prostate, and breast cancer, the same miRNAs target important tumor suppressors - as Phosphatase and tensin homolog (PTEN), p27, p57 and TIMP metallopeptidase inhibitor 3 (TIMP3) - and function as oncogenic miRNAs (27–29).

Long non-coding RNAs (lncRNAs) are non-protein coding transcripts with a minimal length of 200 nts, that are important emerging gene expression regulators. Similar to miRNAs, they are involved in various biological processes including cell differentiation and apoptosis, and in pathophysiological processes as well. Numerous studies highlight that deregulated lncRNAs contribute to hallmarks of cancer including metastasis, drug resistance, and angiogenesis. lncRNAs can exert their roles through different mechanisms that can be summarized as described below (30). Mainly lncRNAs can I) act as transcription factors and modulate gene expression in space and time; II) act as a decoy and remove transcription factors and other proteins away from chromatin, preventing their normal function; III) sequester various microRNAs from mRNA targets, acting as sponges; IV) function as a guide, required for the right localization of factors (including transcription factors and chromatin modifiers) at specific sites for genome regulation; V) act as dynamic scaffolds so that cofactors can assemble together transiently (30).

## MiRNAs and lncRNAs Regulating the GH/IGF1 Axis and IGF System in Cancer: Focus on Pituitary Adenoma, Osteosarcoma, and Colorectal Cancer

A recent review analyzed the current knowledge on miRNAs regulating GH, IGF1, IGF2 and IGF1R in the context of body growth (31). Here we focus on the most recent findings on miRNAs and lncRNAs regulating GH and the IGF system in the context of cancer. In particular, this review will focus on pituitary adenomas, osteosarcoma and CRC which have been documented to be related with GH treatment or GH excess as reported below. Pituitary adenomas, recently reported as pituitary neuroendocrine tumors (pitNETs) (32), are increasingly common intracranial tumors classified as somatotroph adenomas, lactotroph adenomas, corticotroph adenomas, thyrotropinomas, gonadotroph adenomas, plurihormonal adenomas, and non-functioning adenomas (33). Among these, somatotroph adenomas determine supra-physiological GH secretion and cause gigantism/acromegaly (33). The MAPK pathway and the PI3K/AKT pathway are important for pituitary tumorigenesis (34) and are activated also by the GH and IGF signaling. Osteosarcoma is the most prevalent bone malignancy in children and adolescents and is a main cause of cancer-related deaths (35). Interestingly, a recent study reported the overexpression of GHR in osteosarcoma samples and that GHR gene knockdown reduced proliferation, invasion and migration while increasing apoptosis (35). Furthermore, there is a possible increased risk of osteosarcoma in adults who were treated with GH in childhood; bone certainly represents one of the main target tissues for GH action (16, 17). Moreover, both PI3K/AKT and RAS/MAPK signaling pathways, which are key pathways for both GH and IGF signaling, were reported to be over-activated during osteosarcoma progression and metastasis (36, 37). CRC is the third most commonly diagnosed cancer in the world (38) and has a higher prevalence in acromegalic patients with respect to the general population (19). The GH/IGF1 axis and IGF system contribute to promote colorectal tumorigenesis through different mechanisms: deregulating Wnt/β-catenin signaling (39–41), reducing the responsiveness of cells to Transforming Growth Factor Beta (TGFβ) (42), enhancing the anti-apoptotic effects of both Cyclooxygenase-2 (Cox-2) (43) and Bcl2 Like 1 (Bcl-X_L)_ (44), and enhancing the expression of the pro-angiogenetic Vascular Endothelial Growth Factor (VEGF) (45, 46). Moreover, a recent review extensively discussed the role of the activation of PI3K/AKT/mTOR Complex (mTORC) signaling pathway, and Raf/MAPK signaling pathway in the increased glucose uptake and aerobic glycolysis (Warburg effect) by CRC cells (47).

### MiRNAs Regulating the GH/IGF1 Axis and IGF System in Pituitary Adenomas

**Figure 1:**
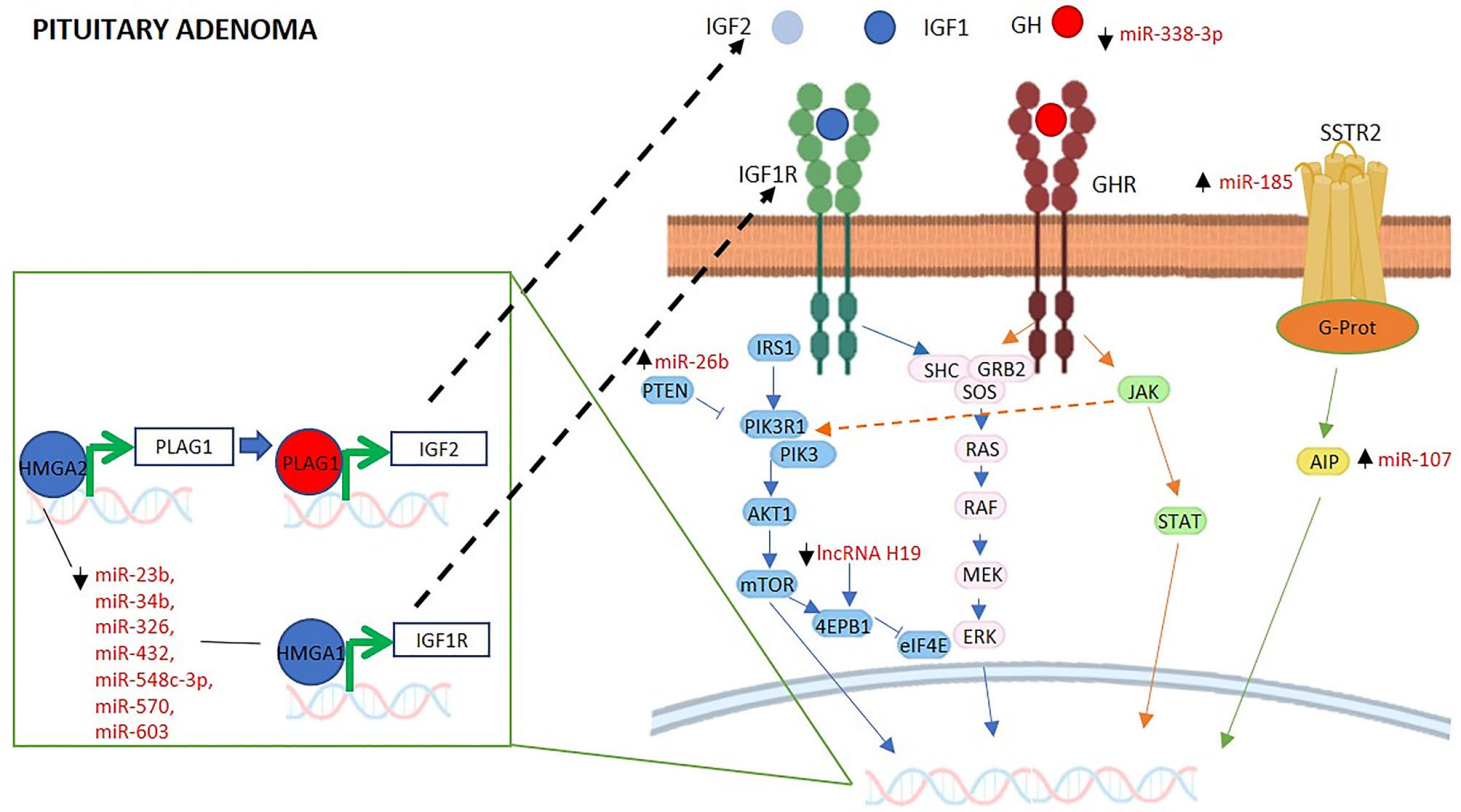
MiRNAs and lncRNAs regulating GHR and IGF signaling in pituitary adenomas. The schematic cartoon describes current knowledge relative to the interactions among miRNAs, lncRNAs and their gene targets within the GH and IGF1 signaling pathways in the context of pituitary adenoma. This figure shows that specific miRNAs and lncRNAs regulate GH and IGF signal transduction by regulating GH secretion and IGF signal transduction downstream their specific receptor. Furthermore, the effect is mediated by miRNA regulation of SSTR2 and several miRNAs are known to regulate in the nucleus the transcription of IGF2 and IGF1R. The miRNAs and lncRNAs known to interact directly with their targets are reported in red. HMGA1, High Mobility Group AT-Hook 1; PLAG1, PLAG1 Zinc Finger; IGF1, Insulin Like Growth Factor 1; IGF2, Insulin Like Growth Factor 2; IGF1R, Insulin Like Growth Factor 1 Receptor; GH, growth hormone; GHR, growth hormone receptor; SSTR2, Somatostatin Receptor 2; IRS1, Insulin Receptor Substrate 1; PTEN, Phosphatase And Tensin Homolog; PIK3R1, Phosphoinositide-3-Kinase Regulatory Subunit 1; PIK3, phosphoinositide 3-kinase; AKT1, AKT Serine/Threonine Kinase 1; mTOR, mammalian target of rapamycin; 4EBP1, Eukaryotic Translation Initiation Factor 4E Binding Protein 1; eIF4E; Eukaryotic Translation Initiation Factor 4E; SHC, Src Homology 2 Domain-Containing protein; GRB2, Growth Factor Receptor Bound Protein 2; SOS, Son of sevenless Ras/Rac Guanine Nucleotide Exchange Factor; RAS, rat sarcoma protein; RAF, Raf Proto-Oncogene, Serine/Threonine Kinase; MEK, Mitogen-Activated Protein Kinase Kinase; ERK, extracellular signal-regulated kinase; JAK, Janus Kinase; STAT, Signal Transducer And Activator Of Transcription; G-prot, G protein; AIP, Aryl Hydrocarbon Receptor Interacting Protein.

Several studies concerning the involvement of miRNAs in regulating the GH/IGF1 axis and the IGF system have been conducted in GH-secreting pituitary adenomas, which are characterized by GH hypersecretion and lead to gigantism in children and acromegaly in adults (48). **Table 1** summarizes the main findings of the studies investigating the role of miRNAs in pituitary adenomas. Of note, the majority of these studies refer to the involvement of miRNAs that target genes tightly related with the GH/IGF1 axis. The following findings are schematically represented in **Figure 1**.

**Table 1:** Current knowledge on the role of miRNAs and lncRNAs on the GH/IGF1 axis and IGF system in pituitary adenomas.

miRNA	Up/down regulation	Target	Condition/effect	Ref.
miR-15a, miR-16-1	↓	GHR (in different cancer cell lines)	Downregulated in GH-secreting and PRLT-secreting pituitary adenomas and in pituitary tumors from MEN1 +/- mice; MEN 1 is a tumor suppressor	Bottoni et al. (49), Lines et al. (50), Elzein et al. (51)
miR-34b, miR-326, miR-432, miR-548c-3p, miR-570, miR-603	↓	HMGA1, HMGA2, E2F1, key proteins in pituitary tumor development	Downregulated in GH-secreting pituitary adenomas; HMGA1 is a positive regulator of IGF1R, IGF1, IGFBP1, IGFBP3; HMGA2 regulates IGF2BP2 thus regulating IGF2 translation	D’Angelo et al. (52), Iiritano et al. (53), Aiello et al. (54)
miR-107	↑	AIP, a putative pituitary tumor suppressor	Upregulated in GH-secreting pituitary adenomas and non-functioning pituitary adenomas. GH3 cells lacking of AIP had increased GH mRNA levels and STAT3 phosphorylation, and showed a slight increase in cell proliferation	Trivellin et al. (55), Fukuda et al. (56)
miR-26b	↑	PTEN, a key tumor suppressor	Upregulated in GH-secreting pituitary adenomas; PTEN activates PI3K/AKT pathway	Palumbo et al. (57)
miR-128	↓	BMI1 which promotes cell proliferation and tumor growth	Downregulated in GH-secreting pituitary adenomas; BMI1 binds to the promoter of PTEN	
miR-23b,	↓	HMGA2	Inhibits cell growth in pituitary adenomas, HMGA2 regulates IGF2BP2 thus regulating IGF2 translation	Leone et al. (58), Li et al. (59), Dai et al. (60), Gan et al. (61)
miR-130b	↓	CCNA2	Inhibits cell growth in pituitary adenomas, CCNA2 promotes cell cycle progression and reduces apoptosis	
miR-185	↓/↑	SSTR2, a G-protein coupled receptor. When activated leads to GH secretion suppression	Downregulated in SSA responder patients and upregulated in SSA non-responder patients with respect to normal pituitaries. Promotes cell proliferation and reduces apoptosis in pituitary adenoma GH3.	Fan et al. (48)
miR-338-3p	↓	GH	Downregulated in GH3 cells, reduced cell proliferation	Lee et al. (62)
	↑	Pttg1	Pttg1 is involved in pituitary adenoma development and invasiveness	Lee et al. (62), Zhou et al. (63)
miR-184	↑	IGF1R (in colorectal cancer)	Upregulated in GH-secreting pituitary adenomas with respect to non-functioning pituitary adenomas and prolactin-secreting pituitary adenomas.	He et al. (64), Mao et al. (65), Wu et al. (66)
miR-21-5p	↑	PDCD4 and Smad7	Upregulated in GH-secreting pituitary adenomas from acromegalic patients, has distant effects on promotion of osteoblast proliferation, differentiation and mineralization	Xiong et al. (67)
**lncRNA**				
H19	↓	4E-BP1, a key mTORC1 substrate involved in protein synthesis induced by GH	Downregulated in tissues from different pituitary tumor subtypes; its levels negatively correlated with tumor volumes; when overexpressed inhibits cell proliferation and colony formation *in vitro* and reduces xenografts tumor burden *in vivo*	Wu et al. (68)
	↑	/	Upregulated in GH-secreting invasive pituitary adenomas with respect to non-invasive pituitary adenomas	Lu et al. (69)
MEG3	↑	/	Promotes GH hypersecretion in GH-secreting pituitary tumors; it is positively correlated with GH and IGF1 serum levels; it is negatively correlated with tumor size. Gsp, an oncogene, could enhance MEG3 expression and may lead to GH hypersecretion.	Mezzomo et al. (70) Tang et al. (71)

4E-BP1, Eukaryotic Translation Initiation Factor 4E Binding Protein 1; AIP, Aryl Hydrocarbon Receptor Interacting Protein; BMI1, B Lymphoma Mo-MLV Insertion Region 1 Homolog; E2F1, E2F Transcription Factor 1; GH, growth hormone; HMGA1, High Mobility Group AT-Hook 1; HMGA2, High Mobility Group AT-Hook 2; MEN1, menin; mTORC1, Mammalian target of rapamycin complex 1; PDCD4, Programmed Cell Death 4; PI3K, phosphoinositide 3-kinase; PRLT, prolactin; PTEN, Phosphatase And Tensin Homolog; Smad7, SMAD Family Member 7; SSA, somatostatin analogues; SSTR2, somatostatin receptor 2.

The studies are reported in chronological order.

MiR-15a and miR-16-1, which belong to the same cluster of tumor suppressor miRNAs, were found to be down-regulated both in GH-secreting and prolactin-secreting pituitary adenomas (49) and were recently confirmed to be down-regulated also in pituitary tumors isolated from mice heterozygote knockout for the Menin 1 (MEN1) gene, which encodes for the tumor suppressor Menin (50). The role of miR-15a and miR-16-1 in pituitary adenomas is not fully elucidated. Interestingly, miR-16 was reported to target GHR in different cancer cell lines (51). Moreover, miR-16 may target also IGF-1, IGF1R, and IGF2R (51). The role of miR-16 in regulating the GH/IGF1 axis represents a potential key point for further investigations within pituitary adenomas.

A group of miRNAs have been found to be downregulated in GH secreting adenomas leading to the overexpression of proteins involved in pituitary tumorigenesis. Specifically, miR-34b, miR-326, miR-432, miR-548c-3p, miR-570, and miR-603, were found to be down-regulated in GH secreting adenomas leading to increased mRNA and protein levels of their target genes including High Mobility Group AT-Hook 1 (HMGA1), High Mobility Group AT-Hook 2 (HMGA2), and E2F Transcription Factor 1 (E2F1) (52). Of note, HMGA1 and HMGA2 are key proteins in the development of pituitary adenomas; indeed, they induce pituitary cell transformation through the enhancement of the activity of E2F1 and other factors (72). Furthermore, HMGA1 is a positive regulator of IGF1, IGF1R, IGFBP1 and IGFBP3 (53, 54).

A miRNA expression profiling in both sporadic GH-secreting pituitary adenomas and non-functioning pituitary adenomas highlighted that miR-107, a miRNA involved in many cancer types, was described to be upregulated. MiR-107 regulates the expression of the putative pituitary tumor suppressor gene Aryl hydrocarbon receptor-Interacting Protein (AIP) (55). Interestingly, rat pituitary adenoma GH3 cells lacking functional AIP, generated with CRISPR/Cas9 editing, showed a strong increase in GH mRNA levels and secretion, and a slight increase in cell proliferation compared to the parental GH3 cells (56). A further investigation of AIP-disrupted GH3 cells highlighted an increase in phosphorylated-STAT3 expression compared with parental GH3 cells (56). The finding is interesting as GH-induced STAT3 phosphorylation is already known to be related with GH over secretion. In addition, a further study showed STAT3 upregulation was associated with GH hypersecretion in pituitary somatotroph adenomas (73).

MiR-26b and miR-128 were found to be respectively upregulated and downregulated in GH-producing pituitary tumors (57). Specifically, miR-26b targets PTEN a key tumor suppressor which, when inactivated, leads to the activation of the PI3K/AKT pathway promoting oncogenesis. This is of note, as this pathway is also crucial in GH and IGF1 signaling (74). miR-128, targets B-lymphoma Mo-MLV insertion region 1 (BMI1) which promotes cell proliferation and tumor growth and binds to the promoter region of PTEN thereby suppressing its expression (57). This may suggest a synergistic action between these two miRNAs in contributing to tumor development, which needs further investigation.

MiR-23b and miR-130b were reported to be downregulated and to inhibit cell growth in pituitary adenomas (58). Interestingly, miR-23b targeted HMGA2, which was previously reported to regulate directly IGF2BP2 that enhances IGF2 translation (59, 60). MiR-130b targeted Cyclin A2 (CCNA2), which promotes cell cycle progression and reduces apoptosis (61).

Somatostatin receptor subtype 2 (SSTR2) was found to be increased in adenomas with respect to normal pituitaries while it was decreased in patients non-responding to treatment with long-acting Somatostatin analogues (SSAs) with respect to responders (48) and was involved in GH secretion suppression (75). In silico analysis evidenced that SSTR2 was a putative target of miR-185 and, intriguingly, miR-185 was upregulated in tumor samples from SSA non-responder adenoma patients with respect to controls while it was downregulated in the SSA responder patients (48). This suggests a possible role of this miRNA in regulating drug resistance. Subsequently, miR-185 was validated to target directly SSTR2 in GH3 cells and, consistently, the transfection of miR-185 mimics reduced the expression of SSTR2 while the use of inhibitors increased its levels (48). Furthermore, miR-185 promoted cell proliferation and reduced apoptosis in GH3 cells (48), which is compatible with a pro-oncogenic role of this miRNA in pituitary adenoma cells.

MiR-338-3p inhibition reduced both GH mRNA and protein levels in GH3 cells and reduced cell proliferation (62). Conversely, miR-338-3p overexpression did not change GH mRNA and protein levels but increased Pituitary tumor-transforming gene 1 (Pttg1) expression, a proto-oncogene which is well known to be related with pituitary adenoma development, invasiveness and recurrence (63). Furthermore, miR-338-3p overexpression reduced GHR mRNA levels as well as the expression of other genes involved in the pathogenesis of pituitary adenomas (62); however, no direct miRNA- mRNA target interactions have been proved so far, therefore further investigations are needed to clarify the role of this promising miRNA in pituitary tumorigenesis particularly concerning its relationship with Pttg1.

A recent study reported different miRNA signatures among non-functioning pituitary adenomas, prolactin-secreting pituitary adenomas and GH-secreting pituitary adenomas compared with normal pituitary glands. In particular, miR-184 was upregulated in GH-secreting pituitary adenomas (64) according to previous reported data (65). Interestingly, miR-184 was reported to inhibit cell proliferation and metastasis in human CRC cells by directly targeting IGF1R (66). This could be of interest considering its role in regulating the IGF1R in several other tumors besides GH-secreting pituitary adenomas.

MiR-21-5p is an oncomiR widely expressed in tumors including GH-secreting pituitary adenomas from acromegalic patients where it was highly expressed compared with patients with non-functioning pituitary adenomas (67). MiR-21-5p has been reported to be one of the most abundant in exosomes derived from GH-secreting pituitary adenoma and downregulated the Programmed cell death 4/Activator protein 1 (PDCD4/AP-1) pathway by targeting PDCD4 and SMAD Family Member 7 (Smad7) (67) with subsequent distant effects that have been shown *in vitro*, specifically osteoblast proliferation, differentiation and mineralization (67). This could represent a novel pathogenic mechanism of particular relevance, especially for acromegaly, and underlines an exosome-mediated communication between GH-secreting pituitary adenomas and both osteoblast proliferation and bone formation.

### MiRNAs Regulating the GH/IGF1 Axis and IGF System in Osteosarcoma

**Figure 2:**
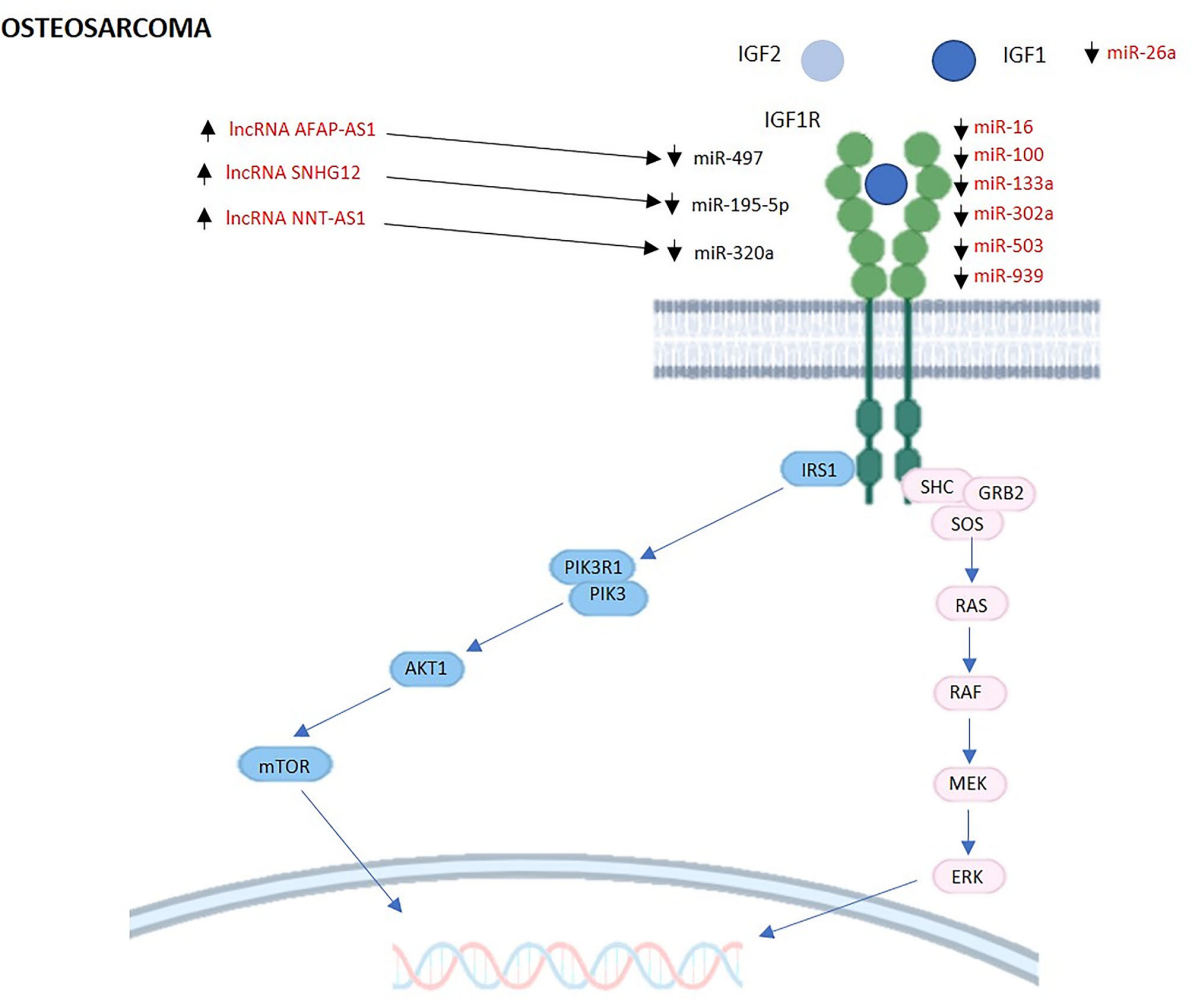
MiRNAs and lncRNAs regulating IGF signaling in osteosarcoma. The schematic cartoon describes current knowledge relative to the interactions among miRNAs, lncRNAs and their targets within the IGF1 signaling pathways in the context of osteosarcoma. The miRNAs and lncRNAs known to interact directly with their targets are reported in red. IGF1, Insulin Like Growth Factor 1; IGF2, Insulin Like Growth Factor 2; IGF1R, Insulin Like Growth Factor 1 Receptor; IRS1, Insulin Receptor Substrate 1; PIK3R1, Phosphoinositide-3-Kinase Regulatory Subunit 1; PIK3, phosphoinositide 3-kinase; AKT1, AKT Serine/Threonine Kinase 1; mTOR, mammalian target of rapamycin; SHC, Src Homology 2 Domain-Containing protein; GRB2, Growth Factor Receptor Bound Protein 2; SOS, Son of sevenless Ras/Rac Guanine Nucleotide Exchange Factor; RAS, rat sarcoma protein; RAF, Raf Proto-Oncogene, Serine/Threonine Kinase; MEK, Mitogen-Activated Protein Kinase Kinase; ERK, extracellular signal-regulated kinase.

Several miRNAs have been reported in the Literature to have a role in the regulation of the GH/IGF1 axis and IGF system in osteosarcoma (**Table 2**). The following findings are schematically represented in **Figure 2**.

**Table 2:** Current knowledge on the role of miRNAs and lncRNAs on the GH/IGF1 axis and IGF system in osteosarcoma.

miRNA	Up/down regulation	Target	Condition/Effect	Ref.
miR-26a	↓	IGF1	When overexpressed inhibits osteosarcoma cell proliferation *in vitro* and tumor growth *in vivo.*	Tan et al. (76)
miR-16	↓	IGF1R	When overexpressed, reduced cell proliferation and tumor growth in mice and inhibited Raf1-MEK1/2-ERK1/2 pathway.	Chen et al. (77)
miR-100	↓	IGF1R	When overexpressed reduced proliferation, invasion and migration *in vitro* and inhibited PI3K/Akt and MAPK/ERK pathways	Liu et al. (78)
miR-133a	↓	IGF1R	When overexpressed decreased cell proliferation, invasion and migration *in vitro*. The injection of osteosarcoma transfected cells into nude mice both subcutaneously and systemically decreased tumor size and lung and liver metastases number respectively.	Chen et al. (79)
miR-503	↓	IGF1R	When overexpressed inhibited proliferation and invasion. MiR-503 and IGF1R expression levels were inversely correlated in osteosarcoma tissues.	Wang et al. (80)
miR-302a	↓	IGF1R	When overexpressed reduced migration and invasion of osteosarcoma cells and was miR-302a expression was correlated with the presence of metastases in patients with osteosarcoma	Zhang et al. (81)
miR-939	↓	IGF1R	Reduces cell proliferation, migration and invasion and increases apoptosis. Deactivated the PI3K/AKT pathway. *In vivo*, decreases tumor growth and weight.	Zhao et al. (82)
**lncRNA**				
NNT-AS1	↑	miR-320 thus indirectly increasing osteosarcoma-promoting genes including IGF1R	When overexpressed, increases cell proliferation, survival and motility *in vitro* and tumor formation *in vivo* while its knockdown increases apoptosis	Li et al. (83)
SNHG12	↑	miR-195-5p thus indirectly increasing IGF1R expression, a miR-195-5p target gene	When silenced, reduces tumor weight and volume in nude mice and reduces osteosarcoma cell growth	Xu et al. (84)
AFAP-AS1	↑	miR-497 thus indirectly increasing IGF1R, a miR-497 target gene	When silenced, reduces proliferation, colony formation, migration and invasion while increases apoptosis rate *in vitro* and inhibited tumorigenesis *in vivo*.	Fei et al. (85)

AKT, AKT Serine/Threonine Kinase; ERK1/2, Extracellular Signal-Regulated Kinase 1/2; IGF1, Insulin Like Growth Factor 1; IGF1R, Insulin Like Growth Factor 1 Receptor; MAPK, Mitogen-Activated Protein Kinase; MEK1/2, Mitogen-Activated Protein Kinase Kinases 1/2; PI3K, Phosphoinositide 3-Kinase; Raf1, Raf-1 Proto-Oncogene, Serine/Threonine Kinase; RUNX2, RUNX Family Transcription Factor 2.

The studies are reported in chronological order and grouped based on target genes, IGF1 and IGF1R.

MiR-26a was described to be downregulated in osteosarcoma tissues with respect to adjacent normal tissues (76). Consistently, *in vitro* experiments using miRNA mimics and inhibitors evidenced that miR-26a overexpression determined a reduction in cell proliferation whereas miRNA inhibition increased cell proliferation. *In vivo* experiments in mice highlighted that the transplantation of osteosarcoma cells stably overexpressing miR-26a, suppressed tumor growth (76). In line with these findings, injection of miR-26a knocked-down osteosarcoma cells in these mice promoted an increase in tumor growth. Thus, miR-26a could be described as a tumor-suppressive miRNA in osteosarcoma and, interestingly, IGF1 was shown to be a direct target of miR-26a (76).

As reported in the following lines, it is noteworthy that the majority of the studies available in the Literature concerning miRNAs, the GH/IGF1 axis and the IGF system in osteosarcoma are focused on miRNAs that target, both directly or indirectly, the IGF1R both *in vivo* and *in vitro*. In particular, miR-16 was reported to be downregulated in osteosarcoma cell lines and tissues (77). MiR-16 overexpression reduced cell proliferation *in vitro* and tumor growth in mice by targeting directly the IGF1R with subsequent inhibition of the Raf1- Mitogen-Activated Protein Kinase Kinase 1/2 (MEK1/2)-ERK1/2 pathway (77). Interestingly, miR-16 was reported also as downregulated in pituitary adenomas (49). Based on these evidences, one could speculate that miR-16 overexpression could represent a potential therapeutic approach for osteosarcoma, and maybe also for pituitary adenomas.

Similar results were described for miR-100 also, which expression was significantly reduced in osteosarcoma tissues and cell lines (78); the role of miR-100 was investigated generating stable cell lines overexpressing this miRNA and through the subsequent observation of reduced proliferation, invasion and migration. In addition, miR-100 was shown to target directly the IGF1R and to inhibit PI3K/AKT and MAPK/ERK pathways. Interestingly, miR-100 overexpression also increased cell sensitivity to cisplatin and this should be taken into account since cisplatin resistance is one of the major issues in osteosarcoma treatment.

A further study showed decreased miR-133a in osteosarcoma cell lines, and this miRNA was shown to control cell proliferation, migration and invasion *in vitro*, and growth and metastasis *in vivo* (79). Specifically, the *in vitro* experiments showed that miR-133a overexpression decreased cell proliferation, invasion and migration. The injection of osteosarcoma transfected cells into nude mice both subcutaneously, and systemically decreased tumor size, and the number of lung and liver metastases, respectively. Silencing of IGF1R exerted effects similar to miR-133a overexpression suggesting that the tumor suppressive function of miR-133a could depend on the regulation of the IGF1R gene (79). Moreover, IGF1R was validated as a direct target of miR-133a and the ERK/AKT signaling pathway was affected also. The further impact of miR-133a on osteosarcoma progression *in vivo* strongly encourages further studies to evaluate whether it could be a new prognostic biomarker and a possible anti-metastatic target for osteosarcoma treatment.

MiR-503 was described also to be significantly downregulated both in osteosarcoma tissues and cell lines when compared to matched adjacent non-tumorous tissue and normal osteoblast cells, respectively (80). A thorough analysis showed that miR-503 overexpression inhibited significantly proliferation and invasion and decreased IGF1R protein levels. IGF1R was subsequently identified as a putative target of miR-503 *via* bioinformatic analysis and, then experimentally validated *via* western blot analysis and luciferase assay. The direct interaction between miR-503 and IGF1R was further confirmed by transfecting overexpressing miR-503 cells with an IGF1R plasmid. The increased IGF1R protein determined a significant increase in proliferation and invasion, further confirming the anti-proliferative and anti-invasive effects of this miRNA. Consistently, an inverse correlation between miR-503 and IGF1R levels was identified in osteosarcoma tissues (80). This study evidenced also that miR-503 levels were lower in osteosarcoma advanced stages compared with initial stages, and this could be helpful in the future to discriminate between different tumor stages also in clinical practice.

A recent investigation reported that a further miRNA, miR-302a, was reduced both in osteosarcoma tissues and cell lines, and a reduction in its expression levels was observed also in metastatic tissues (81). *In vitro*, the overexpression of this miRNA reduced migration and invasion of osteosarcoma cells transfected with a miRNA mimic. IGF1R was validated as a target gene for miR-302a and consistently, IGF1R silencing determined similar effects as miR-302a overexpression (81). This investigation highlighted also that miR-302a suppressed osteosarcoma metastases through targeting IGF1R, and this may provide a novel target to prevent metastasis in osteosarcoma patients.

The most recent study described a decreased expression of miR-939 in osteosarcoma tissues which correlated also with clinical stage and the presence of distant metastases in patients (82). Patients with low miR-939 expression showed decreased overall survival compared with patients with high miR-939 expression. Overall, the data ascribed to miR-939 a role of tumor suppressor, which was confirmed in additional functional experiments performed in osteosarcoma cell lines; the restoration of miR-939 expression inhibited proliferation, migration and invasion while increased apoptosis was observed *in vitro*, and reduced tumor growth and weight *in vivo* (82). Bioinformatic analysis identified IGF1R as a putative target for miR-939, which was further confirmed *via* luciferase assay in cell lines and supported by the observation of an inverse correlation between miR-939 and IGF1R levels in osteosarcoma tissues. The authors showed also that miR-939 overexpression downregulated PI3K-AKT pathway downstream the IGF1R and the restoration of miR-939 could represent a potential therapeutic approach for patients with osteosarcoma following the purpose of a miRNA-based targeted anticancer therapy.

All these studies highlight that IGF1R is a common target of most of the miRNAs studied in the context of osteosarcoma and this confirms also the complexity of the miRNA network in the regulation of gene expression. Interestingly, all these studies have reported only downregulation of specific miRNAs which lead to an increased expression of IGF1R in osteosarcoma. However, the evaluation of other targets within the GH/IGF1 axis and the IGF system would be of paramount importance to clarify the implications of miRNAs in osteosarcoma development and to identify new possible therapeutic targets.

### MiRNAs Regulating the GH/IGF1 Axis and IGF System in Colorectal Cancer

**Figure 3:**
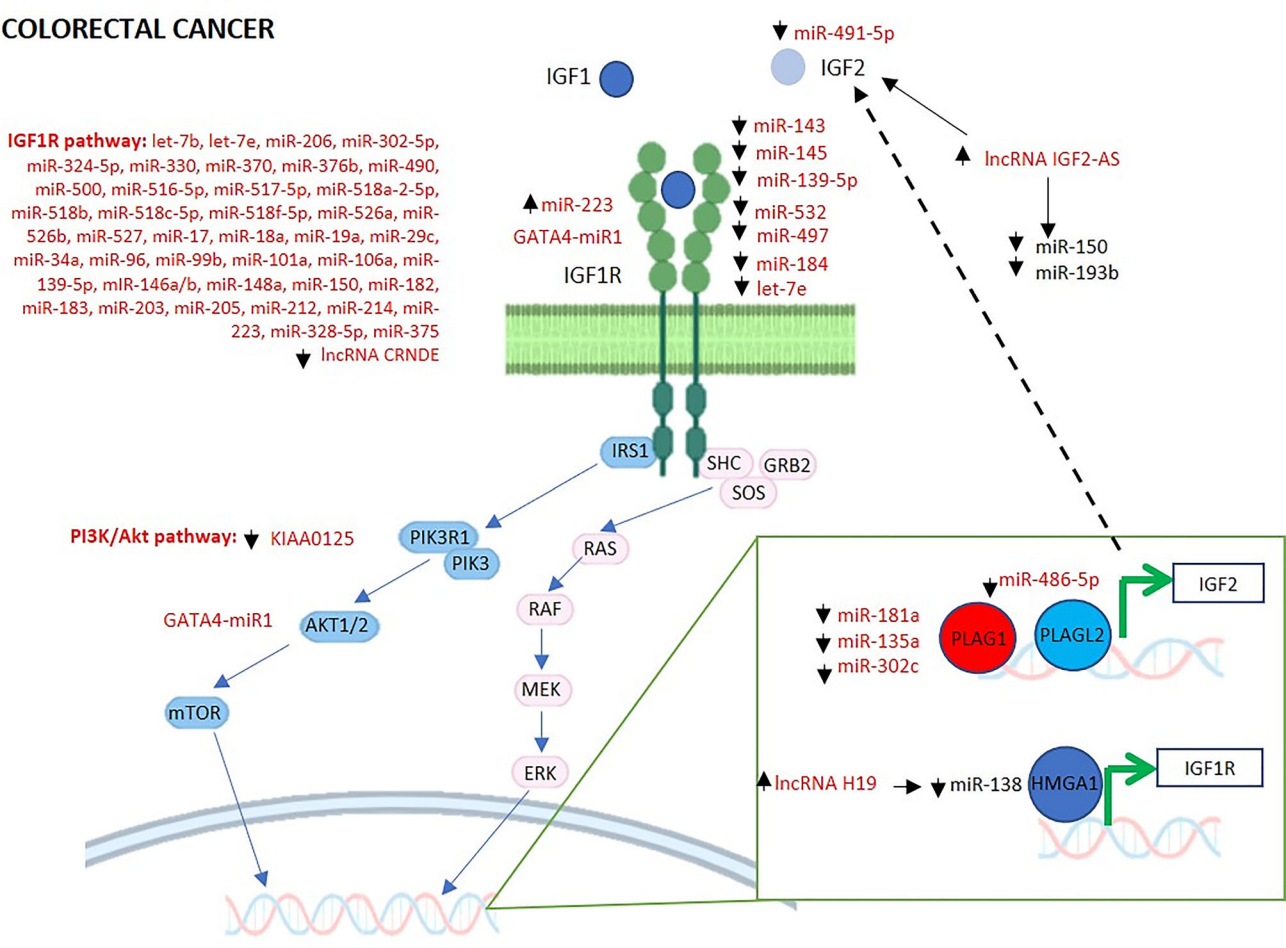
MiRNAs and lncRNAs regulating IGF signaling in colorectal cancer. The schematic cartoon describes current knowledge relative to the interactions among miRNAs, lncRNAs and their targets within the GH and IGF1 signaling pathways in the context of colorectal cancer. The miRNAs and lncRNAs known to interact directly with their targets are reported in red. HMGA1, High Mobility Group AT-Hook 1; PLAG1, PLAG1 Zinc Finger; PLAGL2, PLAG1 Like Zinc Finger 2; IGF1, Insulin Like Growth Factor 1; IGF2, Insulin Like Growth Factor 2; IGF1R, Insulin Like Growth Factor 1 Receptor; IRS1, Insulin Receptor Substrate 1; PIK3R1, Phosphoinositide-3-Kinase Regulatory Subunit 1; PIK3, phosphoinositide 3-kinase; AKT1/2, AKT Serine/Threonine Kinase 1/2; mTOR, mammalian target of rapamycin; SHC, Src Homology 2 Domain-Containing protein; GRB2, Growth Factor Receptor Bound Protein 2; SOS, Son of sevenless Ras/Rac Guanine Nucleotide Exchange Factor; RAS, rat sarcoma protein; RAF, Raf Proto-Oncogene, Serine/Threonine Kinase; MEK, Mitogen-Activated Protein Kinase Kinase; ERK, extracellular signal-regulated kinase.

CRC is one of the most commonly diagnosed malignancies and several studies have investigated the role of miRNAs on the GH/IGF1 axis and IGF system in CRC. The findings are summarized in **Table 3** and schematically represented in **Figure 3**.

**Table 3:** Current knowledge on the role of miRNAs and lncRNAs on the GH/IGF1 axis and IGF system in colorectal cancer.

miRNA	Up/down regulation	Target	Condition/Effect	Ref.
let-7b, let-7e, miR-206, miR-302-5p, miR-324-5p, miR-330, miR-370, miR-376b, miR-490, miR-500, miR-516-5p, miR-517-5p, miR-518a-2-5p, miR-518b, miR-518c-5p, miR-518f-5p, miR-526a, miR-526b, miR-527	↓	IGF1R signaling pathway such as IGF1R, IRS-1, AKT, GSK3 α/β, MEK1, ERK 1/2, p38, p70, p90, ATF-2, and JNK	Downregulated at tumor stages II, III, and IV	Knowlton et al. (86)
miR-21	↑		Upregulated at tumor stages II, III, and IV	
miR-138, miR-143, miR-145, miR-150, miR-192, miR-194, miR-202, miR-320, miR-382, miR-503, miR-519e-5p, miR-526c	↓		Downregulated at all tumor stages	
miR-143, miR-145	↓	IGF1R	Inhibit cell proliferation *in vitro*	Su et al. (87)
let-7e, miR-17, miR-18a, miR-19a, miR-29c, miR-34a, miR-96, miR-99b, miR-101a, miR-106a, miR-139-5p, miR-146a, miR-146b, miR-148a, miR-150, miR-182, miR-183, miR-203, miR-205, miR-212, miR-214, miR-223, miR-328-5p, miR-375	↓/↑	Predicted to target genes involved in PI3K/AKT and IGF-1 signaling pathways	Both up- and downregulated in CRC cells	Josse et al. (88)
miR-223	↑	IGF1R	Inhibits AKT phosphorylation and IGF1R expression in CRC cells	
miR-139-5p	↓	IGF1R	When overexpressed, reduces CRC cell migration and invasion *in vitro* and the formation of tumor metastases in nude mice. Inhibited MEK/ERK/NF-kB signal transduction which is involved in MMP-2 transcription regulation.	Shen et al. (89)
miR-195, miR-497	↓	IGF1R (miR-497)	When overexpressed, miR-497 inhibits also the PI3K/AKT pathway downstream IGF1R and reduces cell proliferation, survival, migration and invasion	Guo et al. (90)
miR-424	↑			
miR-184	↓	IGF1R	When overexpressed inhibits cell proliferation, migration and invasion	Wu et al. (66)
miR-137	↓	YB-1 which, in turn, increases IGF1R	Promotes CRC progression. miR-137 levels were negatively correlated with YB-1 levels. YB-1 levels were positively correlated with IGF1R levels.	Chu et al. (91)
miR-532	↓	IGF1R	When overexpressed, inhibits cell proliferation, migration, invasion, colony formation and promotes apoptosis *in vitro*, while reduces tumor growth *in vivo*. MiRNA levels negatively correlate with IGF1R levels. It inhibits of the PI3K/AKT pathway both *in vitro* and *in vivo.*	Song et al. (92)
let-7e	↓	IGF1R, phospho-AKT	When overexpressed reduced proliferation, migration and invasion while increased apoptosis. Let-7e was reduced by IGF1 highlighting a negative feedback between let-7e and IGF1/IGF1R signaling	Li et al. (93)
	↓	IGF1R	When overexpressed, reduces proliferation arresting the cell cycle in the G1 phase.	Samadi et al. (94)
miR-9	↓ under high glucose concentrations	p-IGF1R, cyclin B1, N-cadherin	Promotes cell proliferation, EMT, migration and invasion	Chen et al. (95)
GATA4-miR1	/	IGF1R, AKT 1/2	When overexpressed, increased the sub-G1 cell cycle population and reduced cell survival and proliferation.	Medlej et al. (96)
miR-675	↑	RB IGF1R (in placentas)	Increases proliferation and promotes malignant transformation *in vitro*. H19, the precursor of miR-675 is located closely to the IGF2 locus.	Tsang et al. (97), Ghafouri-Fard et al. (98), Keniry et al. (99),
miR-483	↑	DLC-1, a tumor suppressor	The expression pattern similar to IGF2 probably due to its location within the IGF2 gene; it increases CRC cell proliferation *in vitro* and promotes tumor growth *in vivo*	Cui et al. (100)
miR-486-5p	↓	PLAGL2 leading to the reduction of IGF2 and β-catenin	When overexpressed, it suppresses cell proliferation and motility *in vitro* and reduces growth velocity, weight of tumors and tumor nodules formation *in vivo*	Liu et al. (101)
miR-181a, miR-135a and miR-302c	↓	PLAG1, a paralogue of PLAGL2 which is an IGF2 transcription factor	When restored, reduce cell viability, arrest cell cycle progression and promote apoptosis	Shi et al. (102)
miR-491-5p	↓	IGF2	When overexpressed, it reduces proliferation *in vitro* and tumor growth *in vivo*	Lu et al. (103)
**lncRNA**				
CRNDE	↓ after treatment with insulin/IGF1/IGF2	Insulin and IGF signaling	The inhibition affects the expression of genes related with insulin/IGF signaling through a feedback mechanism	Ellis et al. (104)
H19	↑	eIF4A3 thus controlling the expression of several genes including cyclin D1, cyclin E1 and CDK4	Accelerates cell cycle progression and CRC proliferation.	Han et al. (105)
		miR-138 which targets HMGA1 which is a positive regulator of proteins belonging to the IGF system	Promotes cell proliferation, migration and invasion *in vitro* and tumor growth and metastases *in vivo*	Yang et al. (106)
IGF2-AS	↑	hsa-miR-150 and hsa-miR-193b. miR-150 targets IGF2BP3.	Negatively correlates with both the overall survival of CRC patients and distant metastases	Zhang et al. (107), Liang et al. (108)
KIAA0125	↓	miR-29b-3p and PI3K/AKT signaling pathway	Sponges miR-29b-3p and may promote CRC development through the regulation of PI3K/AKT	Yang et al. (109)

AKT, AKT Serine/Threonine Kinase; ATF-2, Activating Transcription Factor 2; CRC, colorectal cancer DLC-1, DLC1 Rho GTPase Activating Protein; eIF4A3, Eukaryotic Translation Initiation Factor 4A3; EMT, epithelial-to-mesenchymal transition; ERK1/2, Extracellular Signal-Regulated Kinase 1/2; FSCN1, Fascin Actin-Bundling Protein 1; GSK3 α/β, Glycogen Synthase Kinase 3 Alpha and Beta; IGF1, Insulin Like Growth Factor 1; IGF1R, Insulin Like Growth Factor 1 Receptor; IGF2, Insulin Like Growth Factor 2; IGF2BP3, Insulin Like Growth Factor 2 MRNA Binding Protein 3; IRS-1, Insulin Receptor Substrate 1; JNK, c-JUN N-Terminal Kinase; MMP, matrix metallopeptidase; MEK1, Mitogen-Activated Protein Kinase 1; p38, P38 MAP Kinase; p70, P70 Ribosomal S6 Kinase Alpha; p90, protein 90 ribosomal S6 kinase; PI3K, phosphoinositide 3-kinase; PLAG1, PLAG1 Zinc Finger; PLAGL2, PLAG1 Like Zinc Finger 2; RB, retinoblastoma; YB-1, Y-Box Binding Protein 1.

The studies are reported in chronological order and grouped based on target genes, IGF1R and IGF2.

One of the first studies identified a signature of 130 miRNAs differentially expressed in CRC tissue samples with respect to normal tissue. Moreover, the expression was described to change depending on the tumor stage (86). Among these, 19 miRNAs were found to be downregulated at tumor stages II, III, and IV (**Table 3**), while miR-21 was upregulated. MiR-138, miR-143, miR-145, miR-150, miR-192, miR-194, miR-202, miR-320, miR-382, miR-503, miR-519e-5p and miR-526c were described as downregulated in all CRC stages. Interestingly, 94 of these miRNAs targeted genes within the IGF1R signaling pathway, among these IGF1R, IRS-1, AKT, Glycogen Synthase Kinase 3 (GSK3) α/β, MEK1, ERK 1/2, p38, p70, p90, Activating Transcription Factor (ATF)-2, and c-Jun N-terminal kinase (JNK). The analysis of the phosphorylation status of these targeted molecules in the different CRC stages showed increased activation leading to the identification of the IGF system as a key regulator in CRC progression (86). Of note, the cluster miR-143/145 was further reported to target directly the IGF1R in CRC cells, and provided evidence of a cooperative repression of the IGF1R gene expression by the two miRNAs (87). Furthermore, the authors showed that miR-143/145 inhibited CRC cell proliferation *in vitro*, by inhibiting IGF1R expression highlighting their tumor suppressor activity in CRC, the importance of IGF1R in CRC pathogenesis, and underlying a novel regulatory network to fine-tune cell proliferation (87). In view of these considerations, a combined treatment with both miR-143/145 could represent a strategy to restore their levels in those cancers which exhibit a downregulation, to restore their functions.

A further study identified a group of 77 miRNAs that were differentially expressed between early and late time points of inflammation and tumor growth in a mouse model of colitis-associated cancer, histologically and molecularly similar to human CRC (88). Interestingly, an *in silico* analysis highlighted that these miRNAs were predicted to target genes involved in the PI3K/AKT and IGF1 signaling cascades. Among these 77 miRNAs, 24 were reported to be differentially expressed in human CRC also (**Table 3**), highlighting that the identified signature was relevant for human disease (88). Among these miRNAs, miR-223, the most strongly upregulated miRNA in the mouse model, was further investigated in CRC cell lines. This study showed that miR-223 could target directly the IGF1R thus regulating the PI3K/AKT pathway (88). Another miRNA among these, miR-139-5p, was previously described as one of the most downregulated miRNAs in CRC tissues, and *in vitro* studies demonstrated that miR-139-5p overexpression led to a remarkable reduction in CRC cell migration and invasion, without affecting cell proliferation (89). Consistently, miR-139-5p was shown to be able to suppress the formation of tumor metastasis in nude mice (89). In silico analyses identified IGF1R as a relevant target of miR-139-5p, and was subsequently validated. As expected, miRNA overexpression determined a reduction in IGF1R expression and an inhibition in MEK/ERK/Nuclear factor kappa-light-chain-enhancer of activated B cells (NF-κB) signal transduction which is involved in MMP-2 transcription regulation. These miRNAs and especially miR-223 which are involved in PI3K/AKT, IGF1, and MEK/ERK/NF-κB signaling cascades could represent a molecular link between inflammation and cancer. This aspect should be further investigated to clarify whether carcinogenesis could be triggered by inflammation-induced miRNAs. Moreover, these findings suggested that miR-139/IGF1R regulation was involved in the control of invasive and metastatic capabilities of tumor cells.

A screening of 939 miRNAs highlighted, with respect to the above profiling studies, the downregulation of miR-195 and miR-497 and the upregulation of miR-424 in CRC tissues with respect to non-cancerous mucosa (90). These results were confirmed *in vitro* in CRC cell lines observing reduced proliferation, cell survival, migration and invasion. These three identified miRNAs belong to a cluster of miRNAs (miR-15/16/195/424/497) predicted to target IGF1R. The authors showed that only miR-497 targeted directly the IGF1R. Consistently, mir-497 overexpression inhibited also PI3K/AKT downstream the receptor (90). Reduced ERK activation was also detected suggesting a role of miR-497 in MEK/ERK signaling (90). This highlights the importance of miR-15/16/195/424/497 cluster also in the context of CRC besides pituitary adenoma and osteosarcoma, which were previously described. Moreover, the downregulation of miR-195 and miR-497 and the upregulation of miR-424 in CRC tissues seem to be early events in CRC development, and thus could be studied as potential biomarkers for CRC diagnosis. Furthermore, the restoration of miR-497 levels could be studied as a potential strategy to inhibit IGF1R in CRC.

An additional miRNA identified as having a tumor suppressor role in CRC was miR-184, which levels were reduced in both CRC tissues and cell lines (66). Experimental investigations using miR-184 mimic, showed that the overexpression of miR-184 in CRC cell lines inhibited cell proliferation, migration and invasion. Bioinformatics and laboratory analyses validated IGF1R as a direct target of miR-184. Consistently, IGF1R small interfering RNAs reproduced the actions of miR-184 in CRC (66). MiR-184 could be further investigated as a potential target for CRC treatment due to its role in the regulation and inhibition of IGF1R expression.

A recent study showed that KRAS-mutated CRC cells activated IGF1R expression *via* a novel MEK-DNMT1 signaling pathway involving miR-137 (91). MiR-137 was silenced in both cell lines and tissues indicating that it could act as a tumor suppressor, and its silencing represented an early event during colorectal carcinogenesis (91, 110). In particular, the study by Chu et al. highlighted that KRAS mutation increased IGF1R expression at both the mRNA and protein levels through Y-box-binding protein-1 (YB-1) upregulation which was then validated as a miR-137 target gene. YB-1 is a protein involved in the regulation of genes related with tumor progression. *In vivo*, miR-137 expression was found to be reduced in CRC liver metastases from KRAS-mutated CRC patients, whereas YB-1 and IGF1R were upregulated, confirming the previous data. Furthermore, miR-137 levels were negatively correlated with YB-1 levels, and YB-1 and IGF1R were positively correlated (91). The involvement of miR-137 in the regulation of MEK signaling could open the view for the use of this miRNA as a MEK inhibitor in combination with chemotherapy in patients with KRAS mutant tumors in order to prevent the recurrence of colorectal liver metastases.

Among the deregulated miRNAs in CRC, also miR-532 had a tumor-suppressive role (92). Song et al. showed that miR-532 was downregulated in both CRC tissues and cell lines compared to adjacent normal tissues and normal human colon epithelium, respectively. Further analyses showed that its overexpression inhibited cell proliferation, migration, invasion, colony formation and promoted apoptosis *in vitro*, while reduced tumor growth was observed *in vivo* (92). The authors showed through a luciferase assay that miR-532 targeted directly IGF1R and, consistently, miR-532 expression levels were negatively correlated with IGF1R levels in CRC tissues. Furthermore, IGF1R silencing mimicked the tumor-suppressive effects of miR-532 in CRC cells, further confirming its direct interaction with IGF1R, and miR-532 overexpression inhibited PI3K/AKT pathway activation both *in vitro* and *in vivo* (92). MiR-532 could be a possible non-invasive diagnostic tool due to the specificity of its expression in CRC, and its role in the regulation of IGF1R could offer also an opportunity for CRC treatment.

The let-7 family has been reported to be downregulated in many types of cancer, including CRC (111). IGF1R has been predicted to be a direct target of let-7 miRNAs, and among this family, let-7e had the highest probability score (93, 94). In particular, the study by Samadi et al. showed that let-7e overexpression induced a reduction in IGFR protein content, suppressed growth arresting the cell cycle in the G1 phase, and increased apoptosis. Similar results were reported by Li et al. who showed a reduction in phospho-AKT which is part of the IGF1R signaling pathway (93). In addition, the authors identified IGF1 as one of the predicted targets of the let-7 family, including let-7e. Intriguingly, the treatment of CRC cells with IGF1 reduced let-7e levels, highlighting a negative feedback regulation between let-7e and IGF1/IGF1R signaling (93). This negative feedback regulation could be of great interest in IGF1R-targeted therapeutic strategies in CRC.

Several studies have provided sufficient evidence that miR-9 also participates in colorectal tumorigenesis acting as a tumor suppressor (112, 113). A recent study described a reduction in miR-9 expression in CRC cell lines under high glucose concentrations (95). In particular, miR-9 downregulation due to increased glucose concentration, was associated with increased cell proliferation, epithelial-to-mesenchymal transition (EMT), migration and invasion. It is noteworthy that, when CRC cells were transfected with miR-9 mimic, they expressed less phospho-IGF1R, cyclin B1, and N-cadherin, all involved in cell cycle progression while E-cadherin expression was increased; this latter is involved in cell-cell adhesion and tumor suppression (95). This study suggests that hyperglycemia can modulate tumorigenesis in a way that can be mediated by miR-9 and the control of blood glucose concentrations could serve as an adjuvant potential strategy for CRC treatment.

The newly discovered GATA4-miR1 was located in the second intron of the GATA4 gene, which belongs to a transcription factor family involved in carcinogenesis (96). Bioinformatic tools and experimental research identified IGF1R, AKT1 and AKT2 genes as GATA4-miR1 targets. Consistently, GATA4-miR1 overexpression led to a significant reduction in IGF1R and AKT1/2 expression levels in several cell lines, including the CRC SW480 line. Moreover, a luciferase reporter assay documented a direct interaction between this miRNA and the target genes. GATA4-miR1 overexpression in the CRC cell line determined an increase in the sub-G1 cell cycle population, and a reduction in cell survival and proliferation (96). This newly discovered miRNA warrants further investigation in particular within the context of cell cycle progression and cell proliferation in order to establish whether it could have a therapeutic potential in CRC.

A recent review summarized a number of miRNAs related with IGF2 regulation in CRC (9). MiR-675 and its precursor, the lncRNA H19, were overexpressed in CRC cell lines and tissues suggesting that they might play a role in CRC tumorigenesis. Functional analysis showed that miR-675 downregulated tumor suppressor Retinoblastoma (RB) which controls proliferation and malignant transformation (97). H19 was described to be located close to the IGF2 locus (98) and miR-675 was further reported to target directly the IGF1R in placentas (99). The cross-talk highlighted in this study underlines a relationship between the lncRNA H19 and miR-675 in the pathogenesis of CRC, and underlines the importance of further investigation of the role of lncRNAs and miRNAs in the context of CRC. A further study reported a simultaneous increase in miR-483-3p, miR-483-5p and IGF2 in CRC tissues compared with controls (100). Intriguingly, the authors showed undetectable promoter activity upstream miR-483, which is located within intron 7 of the IGF2 gene. Therefore, it was hypothesized that miR-483 could be co-transcribed with IGF2 due to the lack of a promoter of its own and its overexpression could contribute to the pro-oncogenic effect attributed to IGF2 (100). The authors subsequently proved that miR-483-3p overexpression increased cell proliferation targeting directly DLC-1 which is a tumor suppressor involved in many types of cancer (100). Moreover, from a translational point of view, miR-483-5p could be a feasible biomarker for CRC screening due to the fact that it can detect CRC with an acceptable specificity and sensitivity in serum. A more recent study highlighted that miR-486-5p was downregulated in CRC tissues and cell lines due to higher DNA methylation of its promoter (101). MiR-486-5p overexpression suppressed cell proliferation and motility *in vitro* and reduced growth velocity and weight of tumors and tumor nodule formation *in vivo*. The investigation of the molecular mechanisms underlying these effects showed that miR-486-5p acts as a tumor suppressor by targeting the transcription factor PLAG1 Like Zinc Finger 2 (PLAGL2) which regulates IGF2 and β-catenin expression, thus resulting in the inhibition of cell proliferation and invasion (101). This study highlighted the importance of another epigenetic mechanism, DNA methylation, in the regulation of miRNA transcription which in turn regulate gene expression; this interplay warrants further investigation. Moreover, this study reported increased levels of plasma miR-486-5p in CRC which was tumor derived suggesting it might be explored as a novel prognostic and diagnostic biomarker of CRC. In a specific subtype of CRC, characterized by microsatellite instability, miR-181a, miR-135a and miR-302c were all downregulated in tissue samples and cell lines with respect to microsatellite stable samples (102). MiRNA downregulation was mainly due to the hypermethylation of the CpG island in the promoter region of these miRNAs. The evaluation of their biological functions revealed that they acted as tumor suppressors thereby reducing cell growth and promoting apoptosis. Using a luciferase reporter assay, the authors confirmed that miR-181a, miR-135a and miR-302c targeted directly PLAG1 which is an IGF2 transcription factor (102). The pro-oncogenic action of PLAG1 was mainly mediated by the IGF2 signaling pathway (114), and IGF2 expression was markedly reduced by miR-181a, miR-135a and miR-302c overexpression (102). Interestingly, the restoration of these miRNAs could increase sensitivity to 5-fluorouracil and this should be taken into account in CRC treatment. The most recent study reported that miR-491-5p was downregulated in both CRC tissues and cell lines and its overexpression reduced proliferation *in vitro* and tumor growth *in vivo* (103). Functional analysis demonstrated that miR-491-5p overexpression exerted a tumor suppressor effect by targeting IGF2, and this was also confirmed by the negative correlation between miRNA levels and IGF2 mRNA and protein levels in CRC tissues and cell lines. The miR-491-5p/IGF2 interaction was also confirmed by the finding that IGF2 overexpression partially reverted the anti-tumor effects of miR-491-5p (103). Moreover, this study showed that plasma miR-491-5p was reduced in CRC patients, and thus could have a diagnostic potential in CRC as it can identify CRC with high sensitivity and specificity.

### LncRNAs Regulating the GH/IGF1 Axis and IGF System in Pituitary Adenomas

A recent review summarized the lncRNAs that have been involved in the pathogenesis of pituitary adenomas (115). The findings are summarized in **Table 1** and schematically represented in **Figure 1**. Among these, two studies reported the involvement of the lncRNA H19, an imprinted oncofetal gene, which is aberrantly expressed in cancer tissues (98), and in pituitary adenomas (68, 69). In particular, the study by Lu et al. reported that H19 was highly expressed in GH-secreting invasive pituitary adenomas with respect to non-invasive GH-secreting adenomas (69). The study by Wu et al., evidenced that the lncRNA H19 was downregulated also in tissues from different pituitary tumor subtypes with respect to normal pituitary gland; additionally, its levels were negatively correlated with the tumor volumes (68). Further investigations showed that H19 overexpression inhibited proliferation and colony formation *in vitro*, and reduced xenografts tumor burden *in vivo*. The authors attempted also to elucidate the underlying mechanisms highlighting that H19 directly interacted with the Eukaryotic Translation Initiation Factor 4E Binding Protein 1 (4E-BP1), at the TOS motif inhibiting competitively its binding to Raptor. Overall, this disruption inhibited the recruitment of mTORC1 required for mTOR signaling (68) which is needed for the final protein synthesis induced by GH which requires 4E-BP1 and S6 phosphorylation (116). These investigations highlighted the importance of lncRNA H19 to study the invasion capacity of GH-secreting pituitary adenomas but also that it could be used as a potential biomarker of diagnosis and prognosis.

Two studies have reported the involvement of the lncRNA MEG3 in the development of GH-secreting pituitary adenomas (70, 71). LncRNA MEG3 is encoded by a maternally imprinted gene and suppresses cell proliferation (117). In particular, the study by Mezzomo et al. highlighted a higher expression of MEG3 in GH-secreting pituitary adenomas with respect to non-functioning pituitary adenomas, where the expression was lost in most samples (70). Consistently, the study by Tang et al. reported that MEG3 levels were increased in GH-secreting pituitary tumors with respect to non-functioning pituitary tumors (71). Moreover, the authors reported that MEG3 levels were positively correlated with GH and IGF1 serum levels, whilst they were negatively correlated with tumor size. Interestingly, MEG3 levels were significantly increased in the Gsp-mutated group compared to the non-mutated one, and determined a reduction in tumor proliferation and invasiveness. Gsp is an oncogene which promotes the phosphorylation of cAMP responsive element binding (CREB) and may lead to GH hypersecretion. The proposed mechanism involves Gsp which enhances MEG3 expression by means of phospho-CREB overexpression leading to a reduction in cell proliferation and invasiveness. Indeed, a cAMP responsive element is located at the MEG3 proximal promoter region and is critical for MEG3 activity (71). This study reveals a new possible pivotal gene in the development of pituitary tumors, and this could be useful in the development of novel therapeutic strategies against pituitary adenomas. However, the mechanism underlying the putative tumor suppressive role of MEG3 in GH-secreting pituitary adenomas needs further investigation.

### LncRNAs Regulating the GH/IGF1 Axis and IGF System in Osteosarcoma

To the best of our knowledge only three studies have described the role of lncRNAs targeting the GH/IGF1 axis and the IGF system in osteosarcoma. However, these studies which are summarized in **Table 2** and schematically represented in **Figure 2**, have certainly paved the way to further investigations.

The first study reported in the Literature identified the lncRNA NNT-AS1 (83). In particular, its overexpression increased cell proliferation, survival and motility *in vitro* and tumor formation *in vivo*. Accordingly, NNT-AS1 knockdown determined opposite effects and increased apoptosis. The authors demonstrated that NNT-AS1 acted by sponging miR-320a thereby increasing the expression of osteosarcoma-promoting genes including IGF1R. Consistently, IGF1R protein levels were increased in osteosarcoma cells overexpressing NNT-AS1, while it was decreased after NNT-AS1 knockdown. Furthermore, functional analyses clearly showed that NNT-AS1 effects could be attenuated by miR-320a overexpression (83). The network above including the lncRNA NNT-AS1, miR-320a and its targets, could be of interest to develop new therapeutic strategies for osteosarcoma treatment.

A recent study reported that the expression of the lncRNA SNHG12 was increased in osteosarcoma cell lines (84). In nude mice, the silencing of this lncRNA reduced tumor weight and volume, and functional analysis showed that lncSNHG12 downregulation suppressed osteosarcoma cell growth *via* the regulation of the miR-195-5p/IGF1R axis. In particular, lncSNHG12 downregulation reversed IGF1R-induced metastases and proliferation in osteosarcoma cells. IGF1R was shown to be a direct target of miR-195-5p that was sponged by lncSNHG12 (84). The identification of the lncRNA SNHG12/miR-195-5p/IGF1R axis underlines the importance of lncRNA SNHG12 in the regulation of tumor progression, and could an interesting way to inhibit osteosarcoma progression and metastases.

In the same year, Fei et al. showed that lncRNA AFAP-AS1 was upregulated in osteosarcoma both in tissues and cell lines (85). Functional evaluation highlighted that AFAP-AS1 knockdown significantly inhibited proliferation, colony formation, suppressed migration and invasion and increased apoptosis *in vitro*. Consistently, AFAP-AS1 knockdown inhibited tumorigenesis *in vivo*. A further deepening of the molecular mechanisms identified miR-497 as a direct downstream target of AFAP-AS1. IGF1R was a predicted target of miR-497, and the authors showed that IGF1R negatively correlated with miR-497 and correlated positively with AFAP-AS1 expression in osteosarcoma tissues. Therefore, AFAP-AS1 exerted tumor-promoting actions regulating IGF1R expression by sponging miR-497 (85). The possible role of AFAP-AS1 as a proto-oncogene could represent a promising therapeutic target for osteosarcoma.

### LncRNAs Regulating the GH/IGF1 Axis and IGF System in Colorectal Cancer

Recent studies agreed in identifying lncRNA dysregulation as a critical modulator of CRC progression; these findings are summarized in **Table 3** and schematically represented in **Figure 3**.

The lncRNA CRNDE was identified for the first time by Graham et al. who described it to be upregulated in colorectal adenomas and CRC (118). A further study evidenced that the lncRNA CRNDE was regulated by insulin and IGFs, and suggested that CRNDE nuclear transcripts, generated from CRNDE by alternative splicing, played a role in controlling CRC cell metabolism (104). In detail, nuclear CRNDE transcripts were downregulated in CRC cell lines after treatment with insulin, IGF1 and IGF2. PI3K/AKT/mTOR and Raf/MAPK pathways are the main signaling pathways for insulin, IGF1 and IGF2; functional studies showed that CRNDE knockdown affected the expression of genes strongly related with insulin/IGF signaling. Overall, the findings highlighted a feedback regulatory mechanism between CRNDE and insulin/IGF signaling in CRC (104). From a translational point of view, CRNDE transcripts, which are upregulated in early precancerous stages, could be useful as potential diagnostic biomarkers both in tissue and plasma to detect early cancerous tissue degenerations.

A further lncRNA linked to IGF regulation was H19. As previously described, H19 and IGF2 genes are located on the same chromosome (11p15.5), and share the same imprinting regulatory regions (119). Several studies showed that H19 acts as an oncogene in various cancers, including CRC and its upregulation predicts poor prognosis in patients with CRC (120). In particular, its role in CRC was investigated in five recent studies and they all reported H19 upregulation (105, 106, 120–122). However, the exact mechanism through which H19 exerts its action has not been completely understood. According to Han et al., H19 could control the expression of cell-cycle regulatory genes including cyclin D1, cyclin E1, and Cyclin Dependent Kinase 4 (CDK4) by combining with the Eukaryotic Translation Initiation Factor 4A3 (eIF4A3) and hampering the recruitment of eIF4A3 to their transcripts, thus accelerating cell-cycle progression and CRC proliferation (105). A further study identified that the H19/miR-138/HMGA1 axis was implicated in the regulation of migration and invasion in CRC (106). In detail, the authors demonstrated that H19 promoted cell proliferation, migration and invasion *in vitro* and tumor growth and metastases *in vivo* by sponging miR-138 thereby increasing the expression of HMGA1 (106). As previously reported, HMGA1 is a positive regulator of several proteins belonging to the IGF system (53, 54). Most importantly, the involvement of H19 in the promotion of cell migration and invasion evidenced a potential new treatment option for CRC.

It is well known that eukaryotic genomes are transcribed in both sense and antisense directions, producing abundant levels of noncoding RNAs (107). Among these, IGF2-AS was reported as an antisense lncRNA for the IGF2 gene (108). Interestingly, the authors found that IGF2-AS was overexpressed in CRC cell lines and regulated the expression of specific genes by competitive sponging of miR-150 and miR-193b. Remarkably, IGF2BP3 that increases IGF2 translation, was identified among the predicted target genes for miR-150. In the same year, Zhang et al., by using the CRC Cancer Genome Atlas (TCGA) database, reported a negative correlation between IGF2-AS and the overall survival of CRC patients. Similarly, the analysis of 521 samples (480 tumor tissue and 41 adjacent non-tumor tissue samples) extracted from the TCGA database highlighted that IGF2-AS expression was upregulated in CRC and correlated negatively with distant metastases (121). Thus, the lncRNA IGF2-AS could represent a possible prognosis-related biomarker of CRC. The whole transcriptome sequencing of both early-stage and late-stage CRC tissues highlighted key lncRNAs associated with CRC (109). In particular, the authors validated the downregulation of the lncRNA KIAA0125 and MSTRG.35002 both in early- and late-stage CRC tissues compared with normal colon (109). Of note, functional enrichment analysis found that KIAA0125 was significantly enriched in PI3K/AKT signaling pathway which is pivotal in both GH and IGF signaling.

## Summary and Perspectives

This review highlights the role for miRNAs and lncRNAs in regulating genes related with GH and IGF signaling in the context of cancer. In particular, we focused on the role of miRNAs and lncRNAs in pituitary adenomas, osteosarcoma and CRC, which are known to be tightly related with GH and the IGF system. Both miRNAs and lncRNAs are involved in the regulation of gene expression, the former by directly regulating gene expression, the latter by sponging miRNAs and/or acting less frequently as antisense transcripts. This opens the way to an increasingly more complicated but also more comprehensive regulatory network that needs to be further studied both within tumors per se, and as a regulatory process that can be modified by treatments, requiring the use of GH, and IGF1. To date many studies have reported several miRNAs potentially involved in the modulation of the GH and IGF signaling networks, however, it is still difficult to identify the key players of this complex landscape. Novel potential therapeutic targets emerge based on the deregulated non-coding RNAs and their regulated genes, which warrant further research. In particular, miR-15/16/195/424/497 cluster appears to be of pivotal importance for both pituitary adenoma, osteosarcoma, and CRC, thus it should be further explored. Additionally, several miRNAs emerged as potential therapeutic targets, diagnostic biomarkers, and treatment sensitizers. Moreover, lncRNA-H19 is emerging both in pituitary adenomas and CRC as a promising diagnostic and prognostic marker. Most of the studies within these cancers have currently focused mainly on IGF1R, however, the entire GH/IGF1 axis and IGF system should be studied in order to obtain a comprehensive picture within tumors.

## Author Contributions

Conceptualization: MS and CC. Writing-original draft preparation: CC, GR, and SA. Writing-review & editing: SA, CC, CS, and MS. Visualization: CC and GR. Supervision: MS. All authors contributed to the article and approved the submitted version.

## Conflict of Interest

The authors declare that the research was conducted in the absence of any commercial or financial relationships that could be construed as a potential conflict of interest.

## Publisher’s Note

All claims expressed in this article are solely those of the authors and do not necessarily represent those of their affiliated organizations, or those of the publisher, the editors and the reviewers. Any product that may be evaluated in this article, or claim that may be made by its manufacturer, is not guaranteed or endorsed by the publisher.

## Glossary

4EBP1: Eukaryotic Translation Initiation Factor 4E Binding Protein 1
AIP: Aryl Hydrocarbon Receptor Interacting Protein
AKT: AKT Serine/Threonine Kinase
AP-1: Activator protein 1
ATF-2: Activating Transcription Factor 2
Bcl-X_L_: Bcl2 Like 1
BMI1: B Lymphoma Mo-MLV Insertion Region 1 Homolog
Cas9: CRISPR associated protein 9
CCNA2: Cyclin A2
CDK4: Cyclin Dependent Kinase 4
CRC: colorectal cancer
CREB: cAMP response element-binding protein
CRISPR: Clustered Regularly Interspaced Short Palindromic Repeats
DLC-1: DLC1 Rho GTPase Activating Protein
E2F1: E2F Transcription Factor 1
eIF4A3: Eukaryotic Translation Initiation Factor 4A3
EMT: epithelial-to-mesenchymal transition
ERK: extracellular signal-regulated kinase
FSCN1: Fascin Actin-Bundling Protein 1
GATA4: GATA Binding Protein 4
GH: growth hormone
GHR: growth hormone receptor
GHRH: Growth Hormone Releasing Hormone
GRB2: Growth Factor Receptor Bound Protein 2
GSK3 α/β: Glycogen Synthase Kinase 3 Alpha and Beta
HMGA1: High Mobility Group AT-Hook 1
HMGA2: High Mobility Group AT-Hook 2
IGF1: Insulin Like Growth Factor 1
IGF2: Insulin Like Growth Factor 2
IGF1R: Insulin Like Growth Factor 1 Receptor
IGF2R: Insulin Like Growth Factor 2 Receptor
IGF2BP3: Insulin Like Growth Factor 2 mRNA Binding Protein 3
IGFBP: Insulin Like Growth Factor Binding Protein
IRS: Insulin Receptor Substrate
JAK: Janus Kinase
JNK: c-Jun N-terminal kinase
KIT: KIT Proto-Oncogene, Receptor Tyrosine Kinase
KRAS: KRAS Proto-Oncogene, GTPase
lncRNA: long non-coding RNA
MAPK: Mitogen-Activated Protein Kinase
MEK1/2: Mitogen-Activated Protein Kinase 1/2
MEN1: Menin 1
miRNA: microRNA
MMP: Matrix Metallopeptidase
mTOR: mammalian target of rapamycin
mTORC1: mTOR Complex 1
NF-Kb: nuclear factor kappa-light-chain-enhancer of activated B cells
p27: protein 27, encoded by CDKN1B Cyclin Dependent Kinase Inhibitor 1B
p38: protein 38 mitogen-activated protein kinase
p57: protein 57, encoded by CDKN1C Cyclin Dependent Kinase Inhibitor 1C
p70: protein 70 S6 Kinase Protein
p90: protein 90 ribosomal S6 kinase
PDCD4: Programmed Cell Death 4
PI3K: phosphoinositide 3-kinase
PIK3R1: Phosphoinositide-3-Kinase Regulatory Subunit 1
pitNETs: Pituitary neuroendocrine tumors
PLAG1: PLAG1 Zinc Finger
PLAGL2: PLAG1 Like Zinc Finger 2
PRLT: prolactin
PTEN: Phosphatase And Tensin Homolog
Pttg1: PTTG1 Regulator Of Sister Chromatid Separation, Securin
Raf1: Raf-1 Proto-Oncogene, Serine/Threonine Kinase
RAS: rat sarcoma protein
RB: retinoblastoma
RUNX2: RUNX Family Transcription Factor 2
SHC: Src Homology 2 Domain-Containing protein
Smad7: SMAD Family Member 7
SOS: Son of sevenless Ras/Rac Guanine Nucleotide Exchange Factor
SSAs: Somatostatin analogues
SSTR2: Somatostatin Receptor 2
STAT: Signal Transducer And Activator Of Transcription
TGCA: The Cancer Genome Atlas
TGFβ: Transforming Growth Factor Beta
TIMP3: Metallopeptidase Inhibitor 3
UTR: untranslated region
VEGF: Vascular Endothelial Growth Factor
YB-1: Y-box binding protein 1
Wnt: wingless integrated
